# Supplementary material for: Thermal vulnerability of sea turtle foraging grounds around the globe
Source: Commun Biol. 2024 Mar 21;7:347. doi: 10.1038/s42003-024-06013-y (PMC10958041; doi:10.1038/s42003-024-06013-y)
Supplement: Supplementary file 2 — Supplemental Material [file 42003_2024_6013_MOESM2_ESM.pdf]

Supplemental Material

Thermal vulnerability of sea turtle foraging grounds around the globe

Forough Goudarzi[1,*], Aggeliki Doxa[2,3,4], Mahmoud-Reza Hemami[5], Antonios D. Mazaris[2]

1. Department of Biodiversity and Ecosystem Management, Environmental Sciences Research Institute, Shahid Beheshti University (SBU), Tehran, Iran.

2. Department of Ecology, School of Biology, Aristotle University of Thessaloniki, Thessaloniki 54124, Greece.

3. Institute of Applied and Computational Mathematics, Foundation for Research and Technology-Hellas (FORTH), Heraklion, Crete, Greece.

4. Department of Biology, University of Crete, University Campus Vouton, 70013 Heraklion, Greece

5. Department of Natural Resources, Isfahan University of Technology, Isfahan, 8415683111, Iran.

This PDF includes:

Supplementary Figures:

Supplementary Figure 1. Extracted foraging sites based on satellite tracking associated with foraging behavior of seven sea turtles around the globe.

Supplementary Figure 2. Bivariate (Latitude-Area) distribution of sea turtles' hotspots colored by species.

Supplementary Figure 3. The spatial protection of sea turtles' foraging hotspots by marine protected areas (MPAs).

Supplementary Figure 4. Thermal novelty (TNo) within sea turtles' foraging hotspots by 2100 over longitude.

Supplementary Figure 5. Thermal novelty (TNo) by 2100 within foraging hotspots of the seven sea turtle species separately.

Supplementary Tables:

Supplementary Table 1. Characteristics of identified foraging hotspots for sea turtles around the globe.

Supplementary Table 2. Average, min and max sea surface temperatures (SSTs) of the identified foraging hotspots per sea turtle species for the baseline (2000-2014) and the future (2085-2100) time periods.

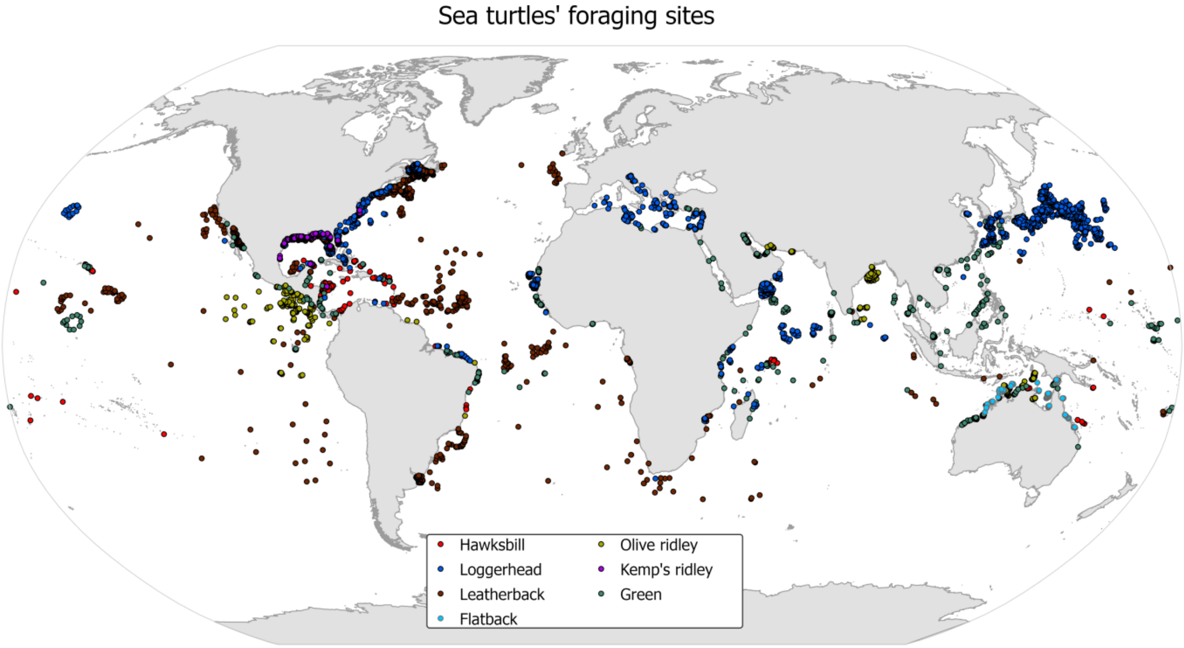

Supplementary Figure 1| Extracted foraging sites based on satellite tracking associated with foraging behavior of seven sea turtles around the globe: of which 262 corresponded to hawksbill turtles, 877 to green turtles, 1407 to loggerhead turtles, 128 to flatback turtles, 1135 to leatherback turtles, 466 to olive ridley turtles, and 542 to Kemp's ridley turtles.

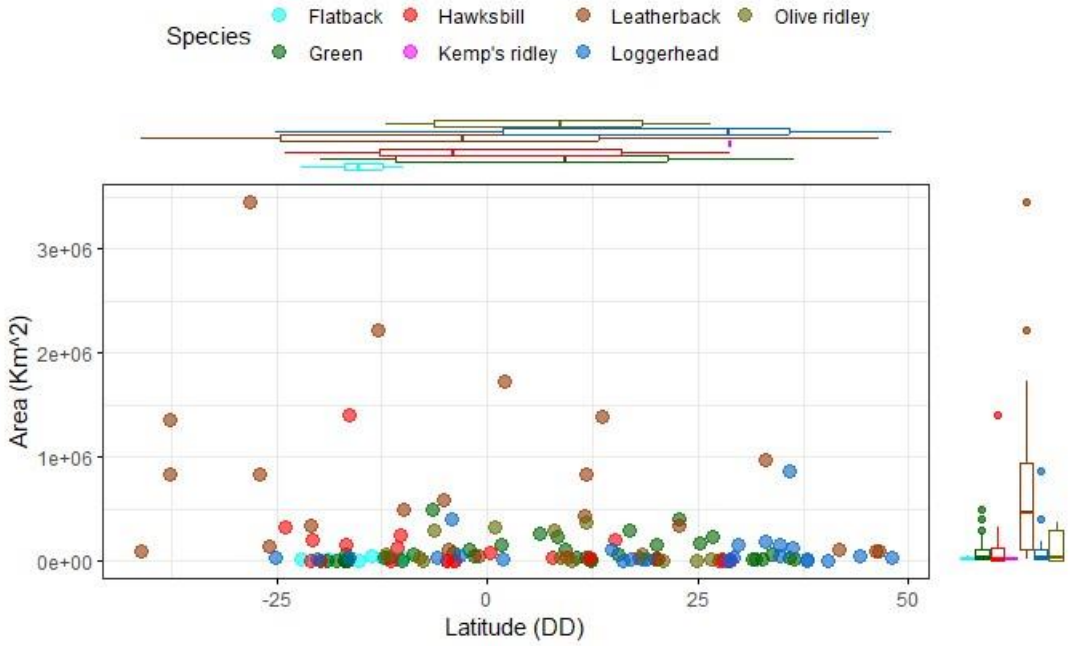

Supplementary Figure 2| Bivariate (Latitude-Area) distribution of sea turtles' hotspots colored by species. Hotspots are mostly less than 100,000 km^2 and distributed mainly between 50° N and 30° S. The marginal boxplots reveal the latitudinal dispersion of hotspots per each species.

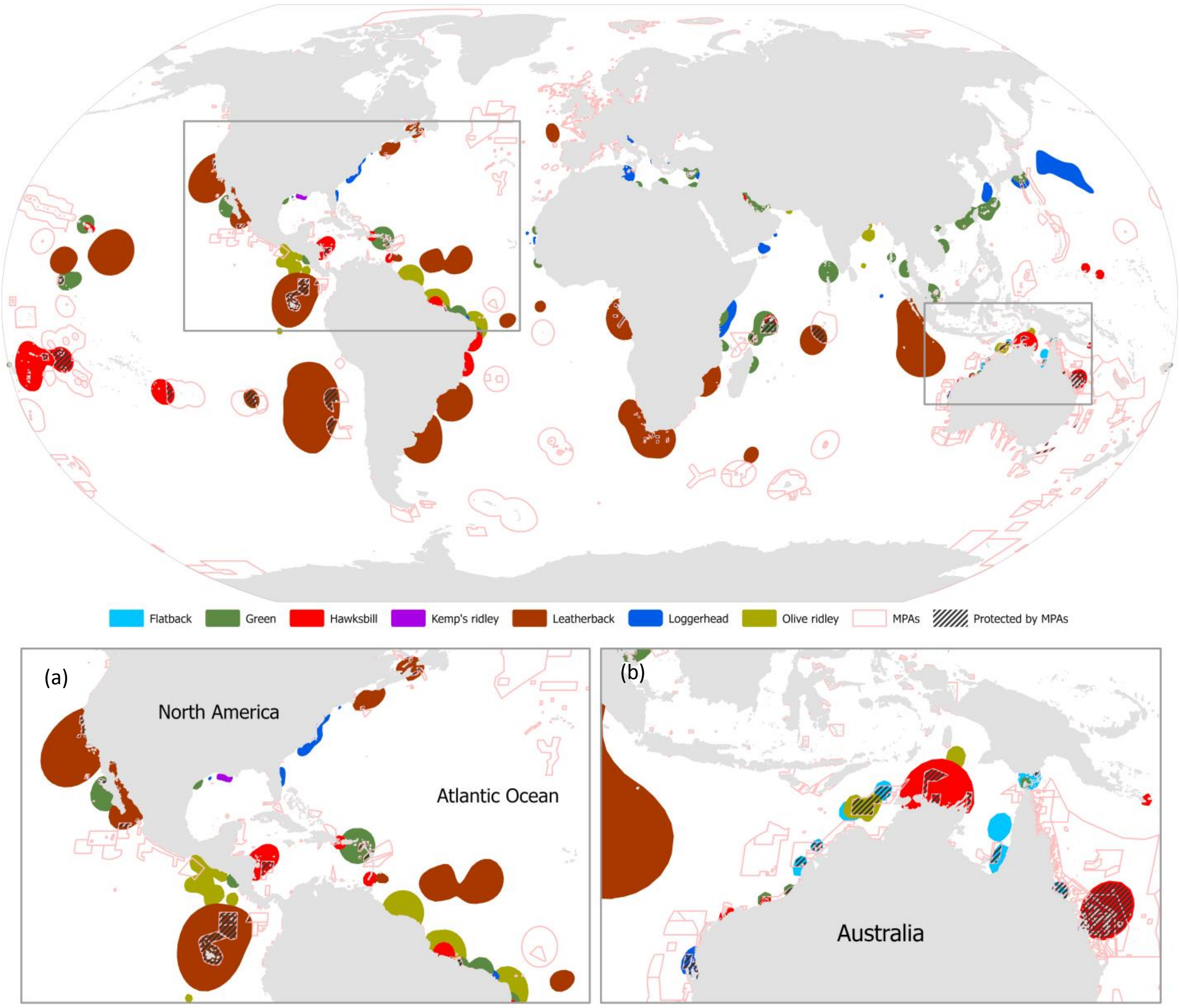

Supplementary Figure 3| The spatial protection of sea turtles' foraging hotspots by marine protected areas (MPAs). 47 out of 133 hotspots are not protected at all, and less than 5% of surface of 34 hotspots is overlapped with MPAs. The two high use regions are shown in panels: (a) the spatial distribution of foraging hotspots in the Gulf of Mexico and (b) the north Australia. Flatbacks' foraging hotspots are mainly (~36%) protected by existing MPAs, but the remaining species have null or very small coverage (0 – 7%) within protected areas. The single foraging hotspot of Kemp's ridley in the Gulf of Mexico is not located within any MPA. The protected parts of hotspots are represented by hatched fill symbol.

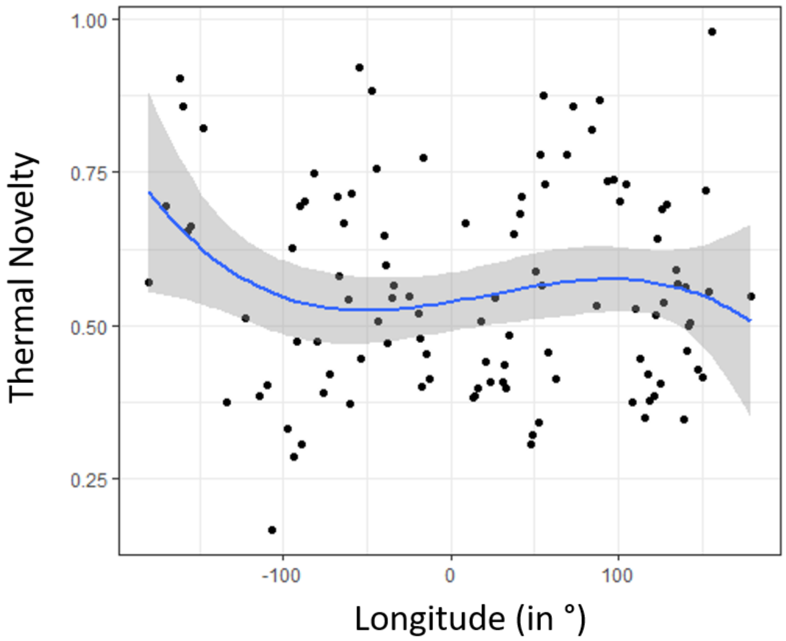

Supplementary Figure 4| Thermal novelty (TNo) within sea turtles' foraging hotspots by 2100 over longitude. The plot was generated using the ggplot package in R, with the lm method and a smoothing span of 5, while the gray zone represents the 95% confidence interval.

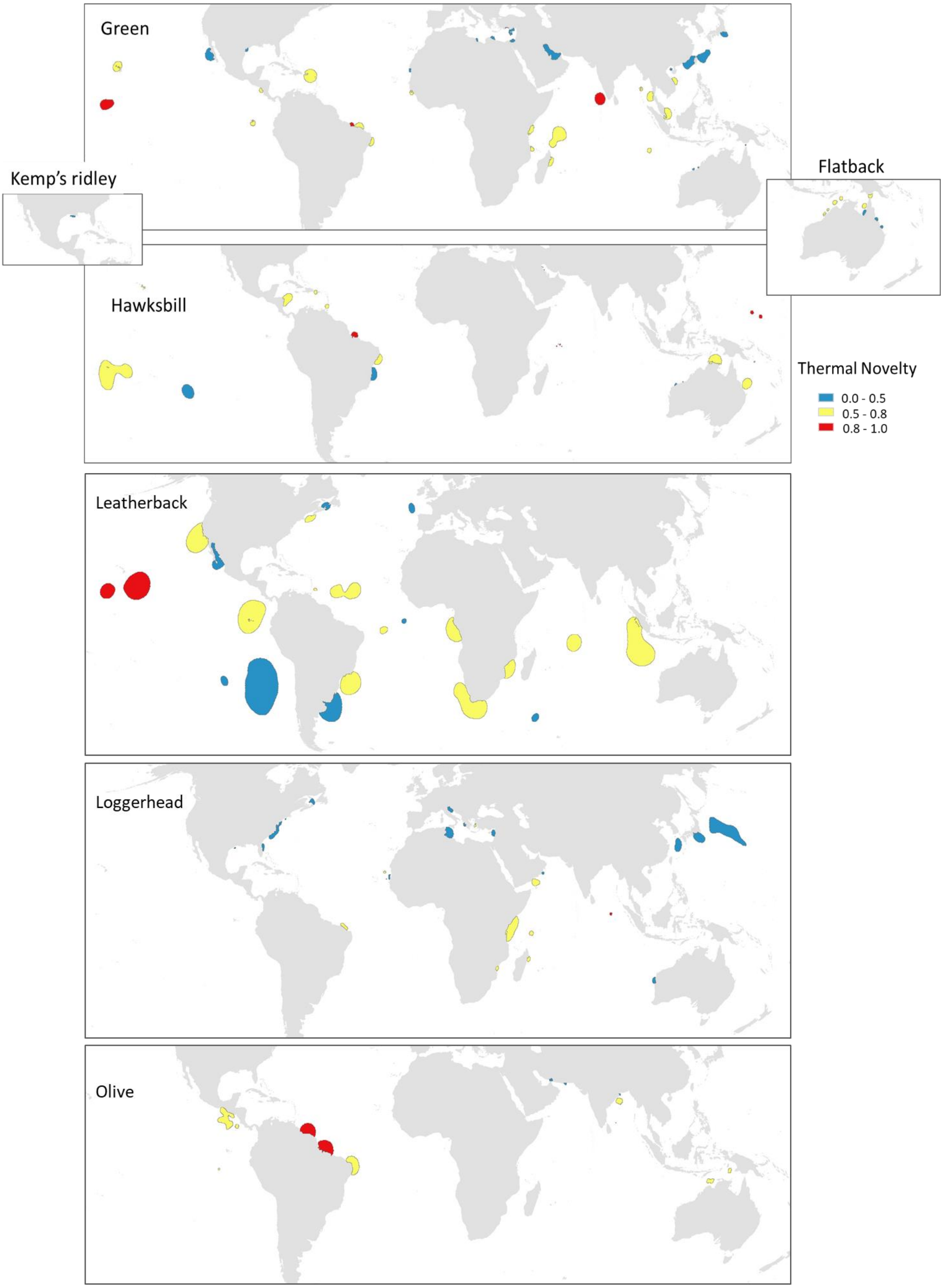

Supplementary Figure 5| Thermal novelty (TNo) by 2100 within foraging hotspots of the seven sea turtle species separately.

Supplementary Table 1| Characteristics of identified foraging hotspots for sea turtles around the globe.

Species	N of hotspot(s)	Area (Km^2)			
		Min	Max	Mean	Sum
Flatback	9	8,253	42,508	23,074	208,426
Green	39	111	490,990	83,358	3,257,646
Hawksbill	24	175	1,398,923	119,111.3	2,868,299
Kemp's ridley	1	-	-	-	15,316
Leatherback	22	17,023	3,438,069	247,248.7	16,483,558
Loggerhead	25	1,688	860,395	94,947.0	2,376,608
Olive ridley	13	1,454	368,274	114,049.4	1,488,770
Total	133				26,698,623

Supplementary Table 2| Average, min and max sea surface temperatures (SSTs) of the identified foraging hotspots per sea turtle species for the baseline (2000-2014) and the future (2085-2100) time periods.

Species	N of hotspot(s)	SST - baseline (◦C)			SST - future (◦C)		
		average	min	max	average	min	max
Flatback	9	27.2	26.0	28.6	30.2	29.0	31.6
Green	39	25.6	23.8	27.2	28.8	27.0	30.4
Hawksbill	24	26.6	25.4	27.9	29.7	28.4	31.0
Kemp's ridley	1	23.1	20.7	25.6	26.7	24.3	29.1
Leatherback	22	21.9	18.7	24.5	25.0	21.8	27.5
Loggerhead	25	22.1	19.5	24.2	25.8	23.2	27.8
Olive	13	27.0	25.6	28.6	30.2	28.7	31.7

To calculate min (respectively max) temperatures, we first estimated minimum (respectively maximum) temperatures at the cell level for each time period and then calculated the average minimum (respectively maximum) temperatures for the ensemble of foraging areas per species and per time period. To calculate average temperatures, we first estimated averages at the cell level by considering both minimum and maximum temperatures for each time period and then estimated the overall average for the ensemble of foraging areas per species and per time period.

We caution that while one may draw general conclusions about the species foraging areas, based on these descriptive statistics of SSTs, the thermal novelty (TNo) analysis is not based on differences in mean values but rather on SSTs distribution dissimilarities between the baseline and the future time periods, thus providing a far higher statistical power in our results. Moreover, the TNo analysis is conducted at the cell level, thus providing accurate estimations for each cell inside each foraging area of each species, whereas the aforementioned SSTs mean and range are estimated at the species level.
