# Supplementary material for: Thermal vulnerability of sea turtle foraging grounds around the globe
Source: Commun Biol. 2024 Mar 21;7:347. doi: 10.1038/s42003-024-06013-y (PMC10958041; doi:10.1038/s42003-024-06013-y)
Supplement: Supplementary file 3 — Description of Additional Supplementary Files [file 42003_2024_6013_MOESM3_ESM.pdf]

Description of Additional Supplementary Files

File name: Supplementary Data

Description: Details on the number of satellite tracks and the extracted points.
