# Supplementary material for: Thermal vulnerability of sea turtle foraging grounds around the globe
Source: Commun Biol. 2024 Mar 21;7:347. doi: 10.1038/s42003-024-06013-y (PMC10958041; doi:10.1038/s42003-024-06013-y)
Supplement: Supplementary file 5 — Reporting Summary [file 42003_2024_6013_MOESM5_ESM.pdf]

Corresponding author(s): DAPR COMMSBIO-23-2556-T

Last updated by author(s): Feb 20, 2024

Reporting Summary

Nature Portfolio wishes to improve the reproducibility of the work that we publish. This form provides structure for consistency and transparency in reporting. For further information on Nature Portfolio policies, see our Editorial Policies and the Editorial Policy Checklist.

Statistics

For all statistical analyses, confirm that the following items are present in the figure legend, table legend, main text, or Methods section.

n/a	Confirmed	
☐	☒	The exact sample size (n) for each experimental group/condition, given as a discrete number and unit of measurement
☒	☐	A statement on whether measurements were taken from distinct samples or whether the same sample was measured repeatedly
☐	☒	The statistical test(s) used AND whether they are one- or two-sided<br>*Only common tests should be described solely by name; describe more complex techniques in the Methods section.*
☐	☒	A description of all covariates tested
☐	☒	A description of any assumptions or corrections, such as tests of normality and adjustment for multiple comparisons
☐	☒	A full description of the statistical parameters including central tendency (e.g. means) or other basic estimates (e.g. regression coefficient) AND variation (e.g. standard deviation) or associated estimates of uncertainty (e.g. confidence intervals)
☐	☒	For null hypothesis testing, the test statistic (e.g. F, t, r) with confidence intervals, effect sizes, degrees of freedom and P value noted<br>*Give P values as exact values whenever suitable.*
☒	☐	For Bayesian analysis, information on the choice of priors and Markov chain Monte Carlo settings
☒	☐	For hierarchical and complex designs, identification of the appropriate level for tests and full reporting of outcomes
☒	☐	Estimates of effect sizes (e.g. Cohen's d, Pearson's r), indicating how they were calculated

Our web collection on statistics for biologists contains articles on many of the points above.

Software and code

Policy information about availability of computer code

Data collection	For identifying foraging grounds, we performed a literature search on Google Scholar using the terms "sea turtle" or "marine turtle", "satellite telemetry" and "foraging". We therefore collected the satellite tracking data reported by all documents published from 1984 to 2020, and extracted 4817 foraging sites (point data).<br>For thermal novelty analysis, sea surface temperature data were retrieved through the World Climate Research Program CMIP6 at the finest possible resolution, at 0.25° using the GFDL-CM4 model. Data are available at the CMIP6 online repository: https://esgf-node.llnl.gov/projects/cmip6/
Data analysis	We used ArcGIS pro 3.0 to digitize the satellite tracking data, and identify foraging sites. To delineate foraging grounds, we conducted kernel density estimation in ArcGIS 10.6.1.<br>Statistical analysis were conducted in R software.<br>The thermal novelty analysis was conducted in R, using the algorithm of Hellinger distance based on Johnson & Watson, 2021. The code is openly available on Github at: https://github.com/smjohnson670/ocean-novelty

For manuscripts utilizing custom algorithms or software that are central to the research but not yet described in published literature, software must be made available to editors and reviewers. We strongly encourage code deposition in a community repository (e.g. GitHub). See the Nature Portfolio guidelines for submitting code & software for further information.

Data

Policy information about availability of data

All manuscripts must include a data availability statement. This statement should provide the following information, where applicable:

- Accession codes, unique identifiers, or web links for publicly available datasets
- A description of any restrictions on data availability
- For clinical datasets or third party data, please ensure that the statement adheres to our policy

All climatic data used at the present study are openly available at https://esgf-node.llnl.gov/projects/cmip6/

Research involving human participants, their data, or biological material

Policy information about studies with human participants or human data. See also policy information about sex, gender (identity/presentation), and sexual orientation and race, ethnicity and racism.

Reporting on sex and gender	NA
Reporting on race, ethnicity, or other socially relevant groupings	NA
Population characteristics	NA
Recruitment	NA
Ethics oversight	NA

Note that full information on the approval of the study protocol must also be provided in the manuscript.

Field-specific reporting

Please select the one below that is the best fit for your research. If you are not sure, read the appropriate sections before making your selection.

☐ Life sciences ☐ Behavioural & social sciences ☒ Ecological, evolutionary & environmental sciences

For a reference copy of the document with all sections, see nature.com/documents/nr-reporting-summary-flat.pdf

Ecological, evolutionary & environmental sciences study design

All studies must disclose on these points even when the disclosure is negative.

Study description	We compiled a global database of satellite tracking data to spatially delineate foraging grounds for the seven sea turtle species and assess their thermal stability by the end of century. We identified 133 foraging areas distributed around the globe, for which only 2% of the total surface is enclosed within an existing protected area.
Research sample	The compiled database included 4817 foraging sites extracted from published satellite tracking data.
Sampling strategy	We performed a literature search on Google Scholar using the terms "sea turtle" or "marine turtle", "satellite telemetry" and "foraging".
Data collection	In total, we scanned and reviewed publications including articles, reports, and gray literature (i.e., reports, theses, and proceedings of conferences) that were published from 1982 to 2020.
Timing and spatial scale	We collected published literature from 1982 to 2020 - Global scale.
Data exclusions	We only selected publications (n=213) that tracked adult turtles between their foraging habitats and breeding sites.
Reproducibility	*Describe the measures taken to verify the reproducibility of experimental findings. For each experiment, note whether any attempts to repeat the experiment failed OR state that all attempts to repeat the experiment were successful.*
Randomization	The study aimed to identify the exact location of sea turtles and their foraging habitats so no randomization process was relevant to the analysis.
Blinding	The study aimed to identify the exact location of sea turtles and their foraging habitats so no blinding process was relevant to the analysis.

Did the study involve field work? ☐ Yes ☒ No

Reporting for specific materials, systems and methods

We require information from authors about some types of materials, experimental systems and methods used in many studies. Here, indicate whether each material, system or method listed is relevant to your study. If you are not sure if a list item applies to your research, read the appropriate section before selecting a response.

Materials & experimental systems

n/a	Involved in the study	
☒	☐	Antibodies
☒	☐	Eukaryotic cell lines
☒	☐	Palaeontology and archaeology
☒	☐	Animals and other organisms
☒	☐	Clinical data
☒	☐	Dual use research of concern
☒	☐	Plants

Methods

n/a	Involved in the study	
☒	☐	ChIP-seq
☒	☐	Flow cytometry
☒	☐	MRI-based neuroimaging
